# Frequency of Th17 CD20+ cells in the peripheral blood of rheumatoid arthritis patients is higher compared to healthy subjects

**DOI:** 10.1186/ar3541

**Published:** 2011-12-15

**Authors:** Paul Eggleton, Edwin Bremer, Joanna M Tarr, Marco de Bruyn, Wijnand Helfrich, Alexandra Kendall, Richard C Haigh, Nick J Viner, Paul G Winyard

**Affiliations:** 1 Peninsula Medical School, University of Exeter, Heavitree Road, Exeter EX1 2LU, UK; 2 Department of Surgery, University Medical Center Groningen, Surgical Research Laboratory, Hanzeplein 1, Groningen 9713 EZ, The Netherlands; 3 Department of Rheumatology, Royal Devon and Exeter Foundation Trust Hospital, Exeter EX2 5DW, UK; 4 Department of Rheumatology, Torbay Hospital, Torquay TQ2 7AA, UK

## Abstract

**Introduction:**

Rheumatoid arthritis (RA) is considered a T cell driven autoimmune disease, therefore, the ability of B cell depleting biologics, e.g., anti-CD20 antibodies, to alleviate RA is unclear. This study examined the proportions of IL-17-secreting lymphocytes in the blood of healthy subjects and RA patients and determined if Th17 cells belong to a CD20+ subset of T cells.

**Methods:**

Fluorescence-activated cell sorting and confocal microscopy verified CD3, CD4/CD8 and CD20-staining of T cells. IL-17 secretion was determined using a commercial assay.

**Results:**

In healthy subjects and RA patients blood, the median percentage of total CD20+ lymphocytes was similar (7.5%; *n *= 6 and 10.3%; *n *= 9, respectively) and comprised predominantly of B cells (~ 86%). However, 2-4% of CD3+ T cells from both healthy subjects (*n *= 7) and RA (*n *= 8) individuals co-expressed CD20. The peripheral blood of healthy subjects contained few IL-17-secreting CD20+ T cells (< 0.1%; *n *= 6). In contrast, in RA blood a median and interquartile range % of, 24.2%; IQR 28.5 of IL-17-secreting T cells were CD20+ (*n *= 9; p = 0.02).

**Conclusions:**

In the blood of RA patients, a greater proportion of Th17 cells are of a CD20+ phenotype compared to healthy individuals. These cells may represent an additional target for anti-CD20 therapies.

## Introduction

Autoimmune diseases such as rheumatoid arthritis (RA) are characterized by chronic inflammation mediated by T and B lymphocytes that accumulate at sites of inflammation (for example, in the synovial joints of patients with RA). Within these sites, a number of subclasses of autoreactive T cells with specific functions (for example, Th1, Th2, or Th17) may play a role in the pathology of the disease and stimulate B-cell proliferation and autoantibody production [1]. In particular, Th17 cells have recently been implicated in the pathogenesis of RA [2].

A cell surface membrane protein, CD20, is found predominantly on B cells, where it functions to aid proliferation and cell cycle progression. Surface CD20 is the target for the biological therapeutic, rituximab (RTX), a chimeric monoclonal antibody [3]. Anti-CD20 therapies have been successful in destroying malignant B lymphocytes expressing this surface marker [4]. More recently, trials to remove circulating B cells from patients with autoimmune disorders such as systemic lupus erythematosus (SLE) and RA have revealed significant clinical activity of anti-CD20 monoclonal antibodies via mechanisms that are not yet completely understood [5-7].

Of note, the anti-CD20-induced depletion of B cells by biologics such as RTX may not be the only mechanism of action accounting for therapeutic efficacy. Indeed, although CD20 is thought of as located primarily on the membrane of B lymphocytes, some studies indicate that a proportion of T lymphocytes also express CD20 [8,9]. This is particularly interesting in view of recent evidence that suggests that specific subsets of T lymphocytes (for example, Th17 cells) may drive the pathological process that is evident in complex autoimmune disorders such as RA. However, the extent and nature of this CD20^+ ^T-cell subset in health and disease-and therefore the possible relevance of this subset in RA-are not yet known. In addition, some groups have suggested that these cells may be T-cell/B-cell doublets [10].

In this study, we confirm that CD20^+ ^T cells are present in the peripheral blood of patients with RA, although the percentage of these cells is small and the proportion of CD20^+ ^T cells in peripheral blood of patients with RA is similar to that of healthy subjects. Importantly, however, we now show that the median percentage of IL-17-secreting cells that are CD20^+ ^T cells is increased by 240-fold in RA patients compared with healthy subjects. Th17 cells are known to play a crucial role in a number of autoimmune diseases, including RA [11]. This finding highlights the possibility that the mode of action of CD20-targeted therapy might include the targeted depletion of CD20^+ ^Th17 cells. We propose that CD20^+ ^Th17 cells may be potential targets for selective depletion in RA.

## Materials and methods

### Patients, healthy subjects, and cell isolation

All diagnoses were made according to the American College of Rheumatology criteria for RA [12]. Nine of the patients were positive for rheumatoid factor or anti-citrullinated peptide antibodies or both. The patients had a mean age of 60.4 years (range of 37 to 100 years), a mean (± standard deviation) disease duration of 19.9 ± 6.8 years, a tender joint count of 2.5 ± 3.3, a swollen joint count of 3.2 ± 3.6, a health assessment questionnaire (HAQ) score of 35.1 ± 17.8, and a disease activity score using 28 joint counts-C-reactive protein (DAS28-CRP) of 3.9 ± 1.3. None of the patients recruited was on anti-tumor necrosis factor or other biological therapeutics. All patients were being treated with methotrexate. Lymphocytes were isolated from the peripheral blood and synovial fluid (SF) of patients with RA and from the peripheral blood of healthy subjects by using Ficoll-Paque Plus (GE Healthcare, Little Chalfont, Buckinghamshire, UK and employing a density gradient centrifugation method in accordance with the instructions of the manufacturer. Flow cytometry analysis was carried out on either total peripheral blood mononuclear cells (PBMCs) or isolated peripheral blood lymphocytes. Cytokine analysis was performed on collected plasma and SFs collected from RA subjects and plasma from healthy subjects. The ethics review board of the Multi-Regional Ethics Committee approved the study (MREC 06/Q2102/56), and all blood samples were obtained with signed consent from the patients and healthy subjects.

### Phenotype analysis of CD20^+ ^and CD20^- ^T cells

After isolating the lymphocytes or PBMCs from both healthy subjects and RA subjects, each subpopulation of lymphocytes was delineated by staining for CD3 (T cells) or CD19 (B cells) by employing phycoerythrin (PE)-conjugated anti-CD3 or PE-conjugated anti-CD19 monoclonal antibodies (BioLegend, San Diego, CA, USA). Then, the number of CD20^+ ^cells in each sample group was determined by co-staining with fluorescein isothiocyanate (FITC)-conjugated anti-CD20 (BioLegend). Appropriately conjugated IgG antibodies-mouse anti-human FITC-IgG2b, K isotype, mouse FITC-IgG1, and PE-mouse anti-human IgG1 (BioLegend)-were used as isotype controls. In a number of healthy subject samples, cells were triple-stained with anti-CD3-FITC, anti-CD20-PE, and anti-CD19-allophycocyanin (APC) or with anti-CD3-CyQ (Cyquant), anti-CD20-FITC, and anti-CD19-PE. Alternatively, cells were stained for CD4-FITC or CD8-FITC, CD19-APC, and CD20-FITC on occasion. To confirm that the anti-CD20-conjugated antibodies were binding specifically to surface-expressed CD20 antigen, isolated PMBCs were washed and pre-incubated with 5 μg/mL RTX (Hoffman-La Roche, Basel, Switzerland) on ice for 15 minutes and washed again before beginning the staining procedures for flow cytometry analysis (Accuri flow cytometer; BD Biosciences, San Jose, CA, USA).

### Cell stimulation and IL-17 generation and detection in CD20^+ ^T cells

IL-17 production in individual cells can be measured in a number of ways, including intracellular cytokine staining (ICS), ELISPOT (enzyme-linked immunosorbent spot), or cytokine capture assays. In the present study, both intracellular and secreted IL-17 IL-17 was assessed. The advantage of the IL-17 capture assay over ICS is that the former monitors only 'live actively secreting cells'. In contrast, intracellular staining techniques allow the monitoring of previously produced IL-17 in fixed cells. The number of IL-17-secreting T cells that were also CD20^+ ^was identified by using an IL-17 cell detection kit (Miltenyi Biotec, Bergisch Gladbach, Germany) and co-staining with anti-CD20-FITC (BioLegend). Briefly, 1.5 × 10^6 ^peripheral blood PBMCs per well were suspended in 150 μL of RPMI and 5% vol/vol autologous serum and 1% glutamine/penicillin/streptomycin and incubated overnight at 37°C, 5% CO_2_/21% O_2_. Then, cells were stimulated with the superantigen CytoStim (20 μL/mL medium; Miltenyi Biotec) for 4 hours at 37°C. Next, the cells were washed several times in RPMI. Cells from unstimulated wells were also collected to serve as negative controls. An IL-17 catch reagent consisting of an anti-IL-17A monoclonal antibody (mouse IgG1) conjugated to CD45-specific monoclonal antibody (mouse IgG2a) was added to label IL-17-secreting cells, followed by 5-minute incubation on ice and 45-minute incubation under slow rotation at 37°C to allow for optimal IL-17 secretion to occur. Cells were subsequently washed and incubated with a 10-μL aliquot of IL-17 detection antibody on ice for 10 minutes. Additional staining antibodies (for example, CD19-FITC and CD4-APC) were added at this stage and analyzed immediately by flow cytometry (Quanta SC flow cytometer; Beckman Coulter, High Wycombe, UK) in accordance with the instructions of the manufacturer. To exclude the possibility of detecting false-positive T cells expressing CD20, by analyzing T-cell/B-cell doublets, stringent gating was performed by using pulse height-versus-width parameter settings. Cells were co-stained with CD19-FITC to ensure that no contaminating B cells were present as either T-cell/B-cell doublets or B cells alone, as assessed with a Quanta SC flow cytometer and Cell Lab Quanta™ software (Beckman Coulter). This particular flow cytometer analyzes cells on the basis of accurate volume/diameter measurements (not forward side scatter, which is an arbitrary measurement of cell size) and side scatter analysis. Therefore, it is possible to gate cells of a specific cell diameter, eliminating the possibility of analyzing T-cell-B-cell aggregates.

For the analysis of surface phenotype and intracellular staining of IL-17, PBMCs were plated into 24-well culture plates (Nunc, Naperville, IL, USA) at 1 × 10^7^/mL in RPMI as described above and cultured overnight at 37°C, 5% CO_2_/21% O_2_. The following morning, aliquots of 10^7 ^cells/mL were stimulated alone with 20 μL of CytoStim for 6 hours at 37°C, with the protein transport inhibitor Brefeldin A being added at a final concentration of 5 μg/mL after 2 hours of this incubation period., For phenotype analysis and intracellular staining of IL-17, aliquots of CytoStim-treated and untreated cells (10^6 ^PBMCs) were transferred to Eppendorf tubes and the cells were washed in cell staining buffer (BioLegend) and stained with mouse anti-human antibodies against CD20-PE, CD19-PE, or IgG1-PE isotype control and then fixed and permeabilized with fixation buffer (BioLegend) and permeabilization buffer (BioLegend), respectively, before being probed for IL-17 with rabbit anti-human IL-17-FITC or IgG1-FITC isotype control antibodies. The cells were spun onto slides and treated with SlowFade Gold antifade reagent with 4',6-diamidino-2-phenylindole (DAPI) (Invitrogen, Paisley, UK). Multiple slides were examined on a Olympus BX60 fluorescent microscope (Olympus, Tokyo, Japan) mounted with a Nikon S10 digital camera (Nikon Corporation, Tokyo, Japan), and images of each experimental condition were acquired.

### Immunoassay of cytokines

Plasma was separated from EDTA (ethylenediaminetetraacetic acid) anti-coagulated peripheral blood and stored at -80°C for later cytokine assays. Plasma and SF cytokine concentrations were analyzed by using commercially available IL-17, IL-21, and IL-23 enzyme-linked immunosorbant assay (ELISA) Quantikine kits (R&D Systems, Inc., Minneapolis, MN, USA). Measurements were performed in duplicate in accordance with the instructions of the manufacturer.

### Confocal microscopy

PBMCs of healthy subjects were stained with anti-CD3-Pacific blue, anti-CD19-APC, and anti-CD20-PE. Subsequently, CD3^+^/CD19^-^/CD20^- ^and CD3^+^/CD19^-^/CD20^+ ^cells were sorted by using a MoFlo high-speed cell sorter (Cytomation, Fort Collins, CO, USA). Sorting was performed at a high purity setting, which restricted the isolation to single cells and excluded doublets. Subsequently, the two cell populations were additionally stained with a combination of anti-CD4-FITC and anti-CD8-FITC. Afterward, staining was analyzed by confocal microscopy (HCX PL APO × 63/1.3 glycerin objective; Leica DM IRE2 Inverted microscope; Leica, Wetzlar, Germany) by using InVivo software (Media Cybernetics, Inc., Bethesda, MD, USA) and a Stanford Photonics XR/Mega-10I (intensified) charge-coupled device (CCD) camera (Stanford Photonics, Inc., Palo Alto, CA, USA).

### Statistical analysis

Data were analyzed by using the Mann-Whitney *U *test. Probability values of *P *less than 0.05 were considered to be statistically significant.

## Results

### CD20^+^/CD3^+ ^lymphocytes are T cells and not T-cell/B-cell doublets

**Figure 1:**
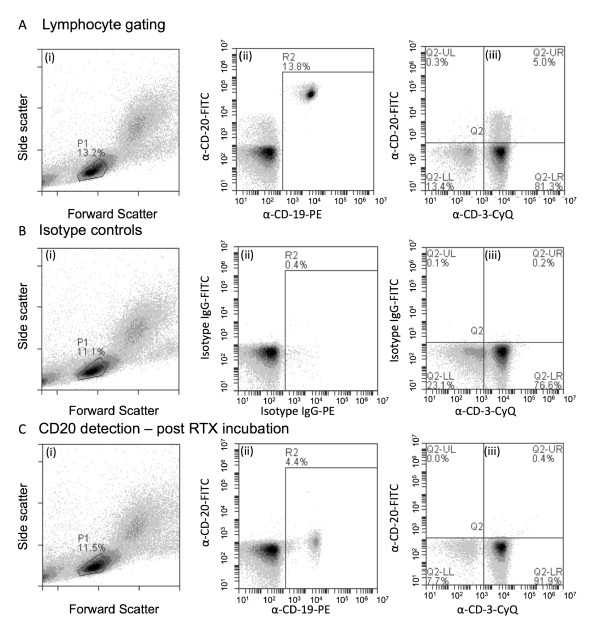
**Gating and control rationale for CD20^+ ^T-cell selection**. **(A) **(i) Typical flow cytometry dot plot of the forward and side scatter of peripheral blood mononuclear cells (PBMCs). (ii) Dot plot of P1 gated cells (as indicated in A) stained with CD20-FITC and CD19-PE and shown in 'R2'. (iii) Dot plot of P1 gated cells, except 'R2', stained with CD20-FITC and CD3-CyQ. **(B) **(i) Typical flow cytometry dot plot of the forward and side scatter of PBMCs. (ii) Dot plot of P1 gated cells stained with isotype control IgG-FITC and isotype control IgG-PE and shown in 'R2' (iii). Dot plot of P1 gated cells, except 'R2', stained with isotype control IgG-FITC and isotype control CD3-CyQ. **(C) **(i) Typical flow cytometry dot plot of the forward and side scatter of PBMCs. (ii) Dot plot of P1 gated cells stained with CD20-FITC and CD19-PE post-RTX treatment and shown in 'R2'. (iii) Dot plot of P1 gated cells, except 'R2', stained with CD20-FITC and CD3-CyQ post-RTX incubation.

**Figure 2:**
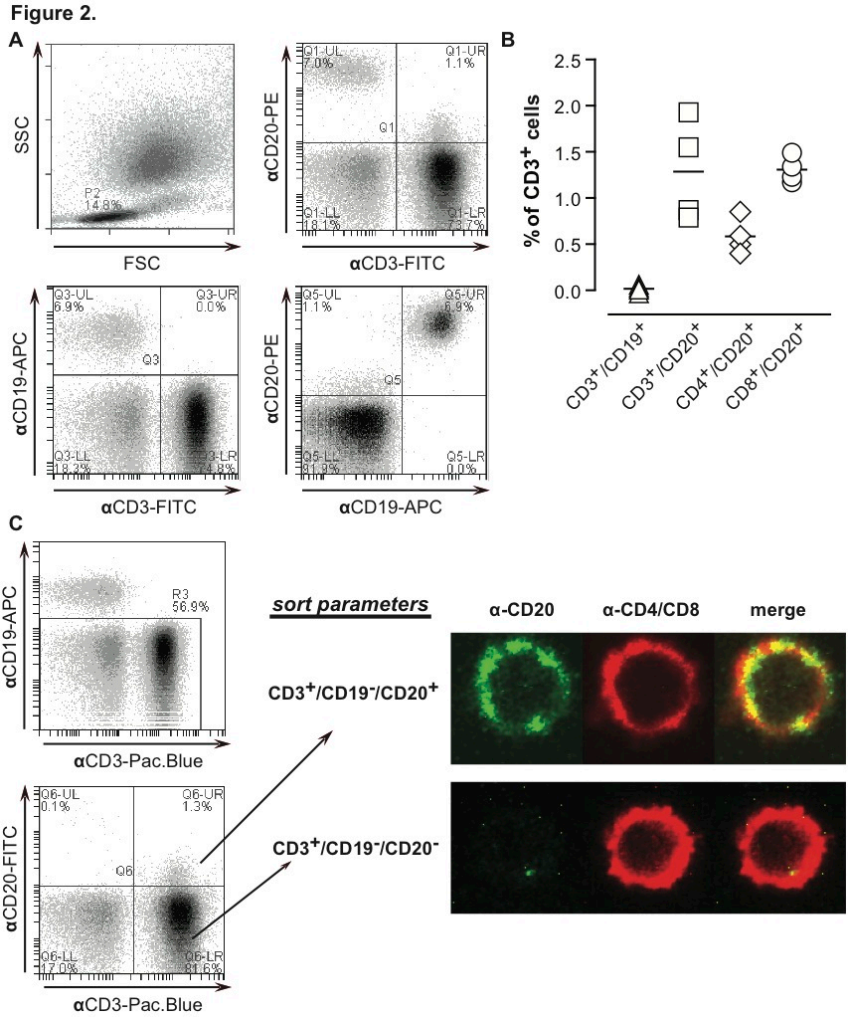
**Detection of subsets of CD3^+^CD20^+ ^T cells in healthy subjects**. **(A) **(i) Typical flow cytometry dot plots of the forward side scatter (FSC) against sideward scatter (SSC) of peripheral blood lymphocytes (PBLs). Within the lymphocyte population, CD3-FITC/CD19-APC/CD20-PE triple-staining was performed on the cells. This staining enabled visualization of (ii) double-positive CD3^+^CD20^+^, (iii) CD3^+^CD19^-^, and (iv) CD19^+^CD20^+ ^events. **(B) **PBLs expressing a CD3^+ ^phenotype (α-CD3-FITC) were gated and evaluated for their expression patterns of CD19 (α-CD19-APC) and CD20 (α-CD20-PE). Alternatively, PBLs were stained with α-CD4-FITC or αCD8-FITC and α-CD19-APC/α-CD20-PE to determine the percentage of CD4^+^/CD20^+^/CD19^- ^and CD8^+^/CD20^+^/CD19^- ^cells. **(C) **Lymphocytes were stained with α-CD3-Pacific blue/α-CD19-APC/α-CD20-FITC, whereupon α-CD3^+^/α-CD19-/α-CD20^+ ^and α-CD3^+^/α-CD19^-^/α-CD20^- ^cells were sorted as illustrated with the sort plots. Subsequently, sorted populations were stained with a mix of α-CD4-PE/α-CD8-PE and the respective populations were analyzed by confocal microscopy for CD4/CD8 (red pseudo color) and CD20 (green pseudo color). Confocal microscopy was performed with a Leica DM IRE2 Inverted microscope (objectives: HCX PL APO 63 ×/1.3 with glycerin; camera: Stanford Photonics XR/Mega-10I (intensified) charge-coupled device (CCD) camera; software: Yokogawa Confocal Scanner Unit CSU10). This experiment was performed on four occasions with similar results. APC, allophycocyanin; FITC, fluorescein isothiocyanate; PE, phycoerythrin.

The possible presence of CD20^+ ^T cells was initially analyzed by performing triple-color flow cytometry for CD3, CD19, and CD20 on PBMCs from healthy volunteers. Briefly, fluorescence analyses were confined to lymphocytes by gating on the lymphocyte population within the forward scatter/sideward scatter dot plot (Figure 1A, gate P1). Within this lymphocyte population, the CD19^+^/CD20^+ ^double-positive B cells were excluded from subsequent analysis (Figure 1A, ii). The remaining lymphocytes were evaluated for CD3 and CD20 expression, which revealed that a small proportion of CD3^+ ^T cells was also dimly positive for CD20 (Figure 1A, iii). The specificity of this staining was verified by performing an isotype control staining, which revealed no non-specific fluorescence (Figure 1B). To further confirm that the CD20 signal detected for the CD3/CD20 double-positive T cells was CD20-specific, PBMCs were pre-incubated with excess anti-CD20 antibody RTX. Subsequent immunofluorescent staining identified that RTX completely abrogated the CD20 staining of CD3/CD20 double-positive staining. In addition, the intensity of the CD20 signal on B cells was diminished by 2-3 log fluorescence intensity (Figure 1C, ii). We performed additional triple-staining for CD3, CD19, and CD20 on peripheral blood lymphocytes of healthy subjects by using flow cytometry (Figure 2A) with and without CD4 and CD8 staining to delineate the subpopulations of T cells showing positivity for CD20 (Figure 2B). In the illustrated flow cytometry plots of a representative sample, approximately 1.5% of CD3^+ ^cells were also CD20^+^, whereas CD3^+ ^cells did not express the B-cell marker CD19 (Figure 2A). In line with earlier findings [9], the staining intensity for CD20 on these T cells was an order of magnitude lower than that on CD20/CD19 double-positive B cells. Within the CD3^+ ^population of four healthy subjects analyzed, the median percentage (interquartile range, or IQR) of cells, which were CD3^+^/CD20^+^, was 1.20% (IQR 1.02), and the ratio of CD8 to CD4 cells was 3:1 (Figure 2B). We performed flow cytometric cell sorting on four healthy subjects to obtain the CD3^+^/CD20^+^/C19^- ^cell population. Subsequent confocal microscopy of these CD3^+^/CD20^+^/CD19^- ^cells (Figure 2C) demonstrated that they also express the T-cell marker CD4 or CD8. Importantly, CD3^+^/CD20^+^/CD19^- ^cells were clearly identifiable as single cells and not, as previously suggested, as T-cell/B-cell doublets [10]. Interestingly, the expression of CD20 on the T cells appears rather patchy and may reflect cross-linking of anti-CD20 to CD20, which has been observed to result in CD20 translocating to lipid rafts [13]. Taken together, these results confirm the presence of CD20^+^/CD3^+ ^T cells in peripheral blood of healthy volunteers.

### The frequency of CD20^+ ^T lymphocytes is not elevated in the blood of RA patients compared with healthy subjects

**Figure 3:**
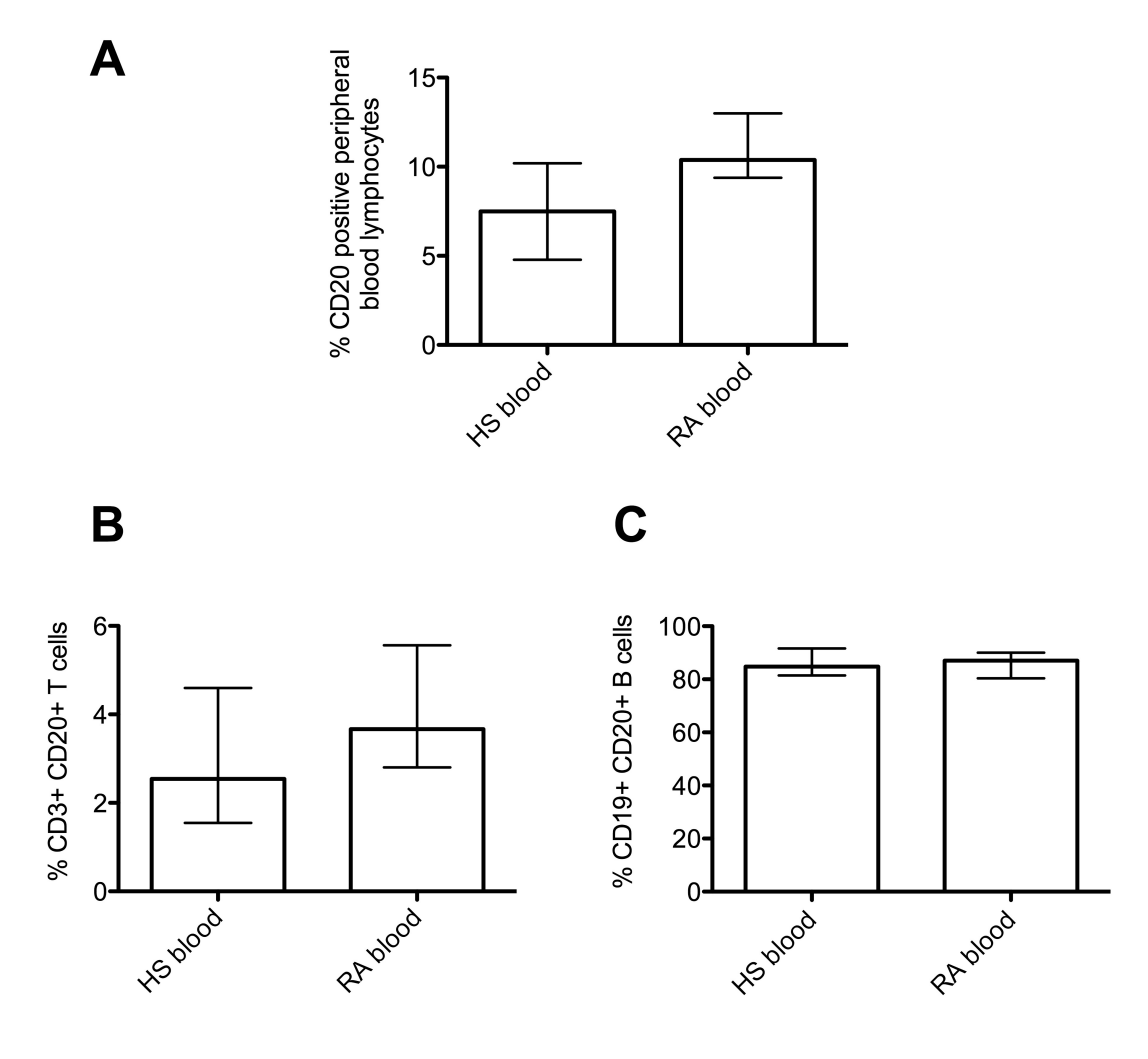
**Expression of CD20 on B and T lymphocytes from the peripheral blood of healthy subjects (HS) compared with rheumatoid arthritis (RA) patients, as obtained by flow cytometry**. **(A) **The percentage of CD20^+ ^peripheral blood lymphocytes in HS and RA patients. **(B) **The percentage CD3^+^CD20^+ ^T cells in HS and RA patients. **(C) **The percentage of CD19^+^CD20^+ ^B cells in HS and RA patients as analyzed by flow cytometry. The bars represent the median percentage and interquartile range. The expression patterns of CD20^+ ^cells represent data from 11 HS and 8 RA blood samples in total.

Initially, we assessed the percentage of CD20^+ ^lymphocytes (B and T cells combined) in the peripheral blood of healthy subjects and RA patients. We observed a similar median percentage of total peripheral blood lymphocytes (approximately 8% to 10%) that were CD20^+ ^(Figure 3A). Next, we determined the relative frequency of CD20/CD3 double-positive T cells in the blood of RA patients and healthy subjects by flow cytometry. The median percentages with IQR, of this cell subset were similar in the peripheral blood of healthy subjects and RA patients (Figure 3B): 2.5% (IQR 3.1) and 3.7% (IQR 2.8), respectively (Figure 3B). In two of these patients, the percentages of CD20/CD3 double-positive T cells were 8.5% and 1.3%, respectively, in their SF and 3.28% and 0.37%, respectively, in their peripheral blood. In addition, the median percentages of CD19/CD20 double-positive B cells present in peripheral blood lymphocytes of healthy and RA subjects were identified by flow cytometry (Figure 3C) and consisted of 84.8% (IQR 10.2) and 87.0% (IQR 9.7) CD19^+^/CD20^+ ^B cells in healthy subjects and RA patients, respectively. Similarly, in the SF available from two patients with RA, the percentages of CD19/CD20 double-positive B cells were 60.0% and 79.0% in the two SF samples.

### The median percentage of IL-17-secreting CD20^+ ^T lymphocytes is significantly elevated in rheumatoid arthritis compared with healthy control subjects

**Figure 4:**
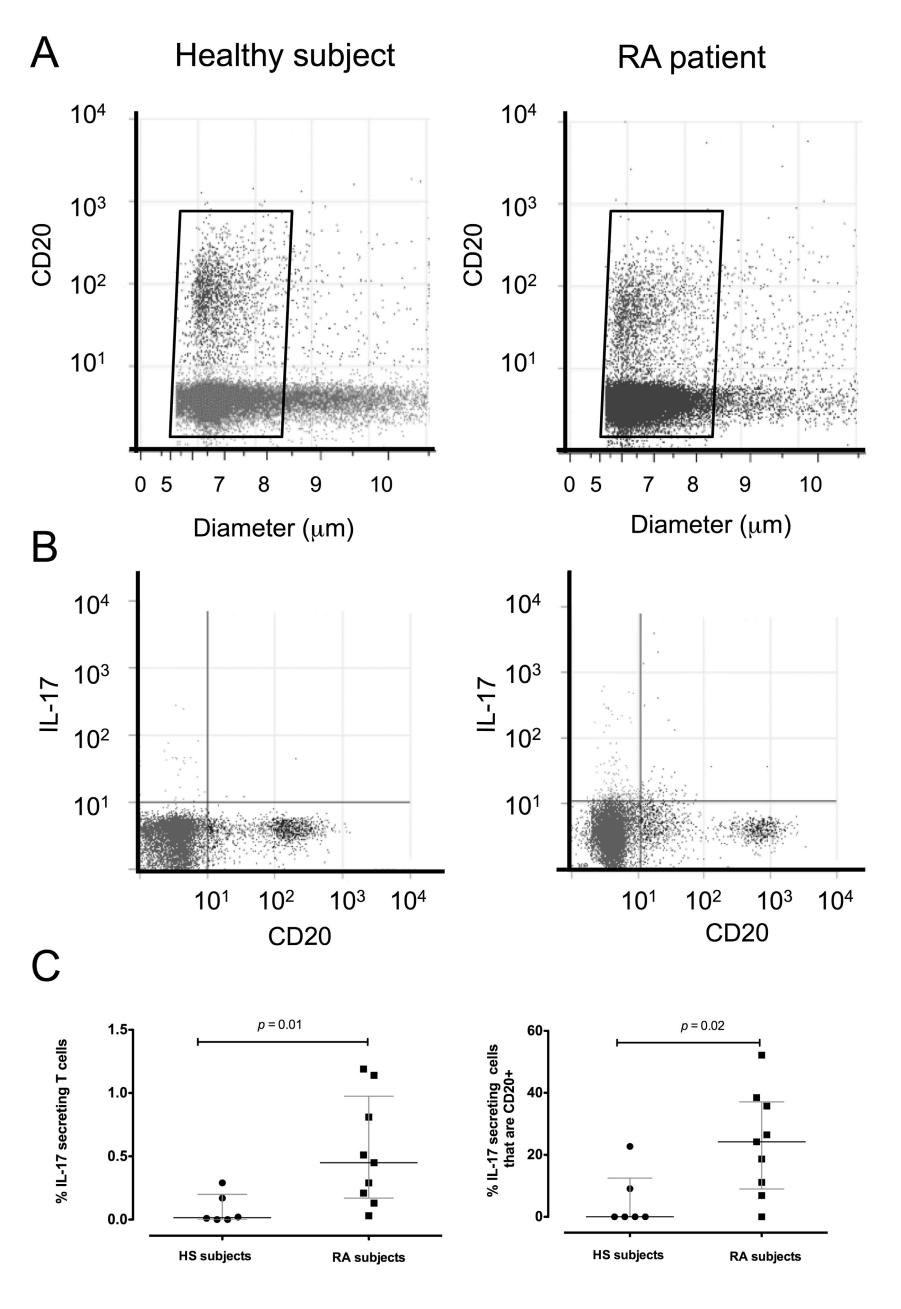
**CD20^+^, IL-17-secreting, and IL-17-secreting/CD20^+ ^lymphocytes in healthy subjects (HS) and rheumatoid arthritis (RA) patients**. **(A) **Dot plot of size versus CD20 positivity in representative peripheral blood lymphocyte (PBL) samples from an HS and an RA patient. **(B) **Typical flow cytometry dot plots showing the proportion of CD20^+ ^and IL-17-secreting lymphocytes in representative PBL samples from an HS (left panel) and an RA patient (right panel). Each dot plot shows the proportion of CD20^+ ^cells (lower right quadrant), IL-17-secreting cells (upper left quadrant), and IL-17-secreting CD20^+ ^cells (upper right quadrant). **(C) **The proportion of PBLs that were IL-17-secreting in HS (*n *= 6) and RA subjects (*n *= 9, left panel) and the proportion of IL-17-secreting cells that were specifically CD20^+ ^T cells (right panel). The horizontal bars represent the median percentage and interquartile range of each group. IL, interleukin.

Since recent studies highlight a possibly central role for IL-17-secreting T lymphocytes in the pathogenesis of RA, we next assessed whether the percentages of IL-17-secreting lymphocytes in the peripheral blood of healthy subjects and RA patients differed. We excluded analysis of doublets in flow cytometry data by gating on CD20^+ ^lymphocytes between 5.0 and 8.5 μm in diameter (Figure 4A). Gated cells were then analyzed for double-staining with CD20 and IL-17 (Figure 4B). IL-17-producing CD19^-^/CD20^+ ^gated lymphocytes were detected in PBMC preparations following 4 hours of CytoStim stimulation. Typical dot plots of IL-17-secreting lymphocytes from a healthy subject (Figure 4B, left panel) compared with a patient with RA (Figure 4B, right panel) are shown.

Others have reported that between 0.04% and 2% of CD4 T cells secrete IL-17 following CytoStim stimulation [14]. In our study, IL-17 was produced by 0.0% to 0.29% of healthy subject PBLs (*n *= 6) and 0.03% to 1.2% of RA patient PBLs (*n *= 9). The median percentages (with IQRs) were 0.02% (0.2) and 0.5% (0.8), in HS and RA respectively. Since about 70% of PBLs in HS and RA blood are T cells, the values in our study are in approximate agreement with another study that looked at T cells directly [14]. As shown in Figure 4C (left panel), there was a 45-fold increase (*P *= 0.01) in the median percentage of IL-17-secreting T cells in the blood of RA patients compared with healthy control subjects. We further looked at the ratio of CD8 to CD4 cells expressing CD20 and secreting IL-17 in a separate cohort of healthy subjects. Among the CD20^+ ^IL-17-secreting T cells, 45% co-expressed CD4 and 55% co-expressed CD8.

**Figure 5:**
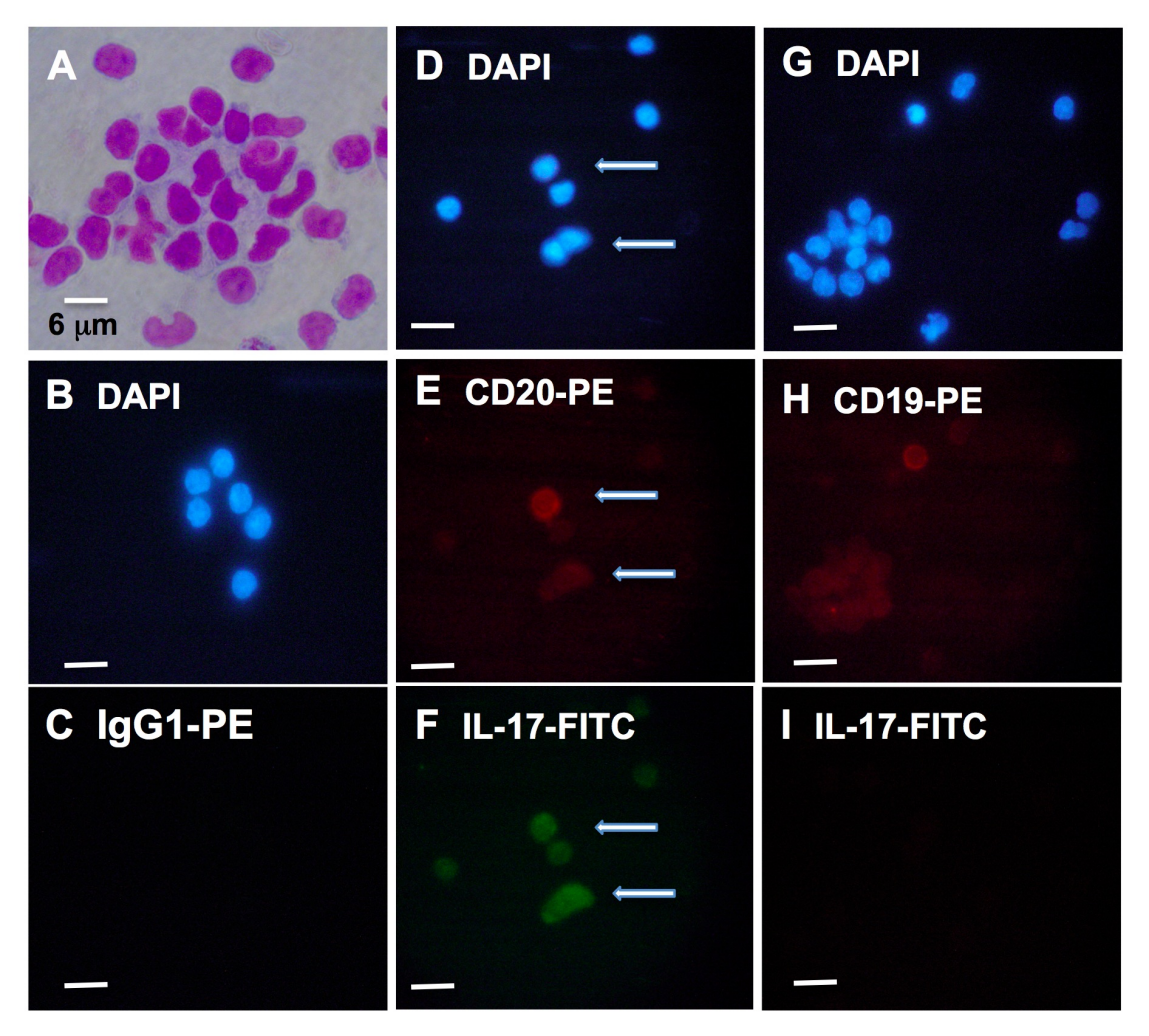
**Immunohistochemical detection of CD20^+ ^lymphocytes with intracellular staining for IL-17**. **(A) **Peripheral blood mononuclear cells were isolated from peripheral blood and stimulated with CytoStim followed by Brefeldin A and stained histochemically with Diff-Quick™. **(B-I) **Cells were then phenotyped for IgG1-PE isotype control (C), CD20-PE (E), or CD19-PE (H). The cells were then fixed and permeabilized and stained for IL-17-FITC, as seen in (C), (F), and (I), respectively. The cells were mounted on slides with antifade fluid supplemented with DAPI for nuclear staining (B, D, and G). Arrows in the middle column of panels depict triple-stained CD20^+ ^(red) lymphocytes, which are IL-17^+ ^(green). The right-hand column of panels depicts CD19^+ ^lymphocytes (red), which are IL-17^-^. Images were viewed at ×1,000 with an Olympus BX60 microscope with C-mount and a Nikon S10 digital camera. The size bar represents 10 μm unless otherwise stated. All specimens were processed at the same time, as described in Materials and methods. At least 10 fields of view were examined for each panel. DAPI, 4',6-diamidino-2-phenylindole; FITC, fluorescein isothiocyanate; IL, interleukin; PE, phycoerythrin.

As we observed a higher percentage of IL-17-secreting T cells in the peripheral blood of RA patients compared with healthy control subjects, we were interested to know what proportion of these cells were also CD20^+ ^T cells. We captured the IL-17-secreting lymphocytes and screened them for CD20 expression by flow cytometry. As shown in Figure 4C (right panel), in patients with RA, there was an increase of two orders of magnitude (*P *= 0.02) in the median percentage of IL-17-secreting cells that were also CD20^+ ^(24.2%) compared with healthy control individuals (< 0.01%). This represented a 240-fold increase in the median percentage of IL-17-secreting cells that were also CD20^+ ^in RA patients compared with healthy subjects. We sought to obtain confirmatory visible evidence of CD20^+ ^cells generating IL-17 directly: PBMCs were stimulated with CytoStim and treated with Brefeldin A (to prevent IL-17 secretion) and triple-stained. Fresh cells were stained with CD20-PE or CD19-PE (for phenotype analysis); fixed, permeabilized, and stained for intracellular IL-17-FITC. Finally, the cells were treated with DAPI (for nuclear staining). As shown in Figure 5, CD20^bright ^cells (middle column, Figure 5D-F) depicted with arrows, were also IL-17^+^. As expected from the flow cytometry analysis above, some CD20^dim ^cells were positive for IL-17 staining. In control experiments, CD19^+ ^cells (right-hand column, Figure 5G-I) were negative for IL-17 intracellular staining.

**Figure 6:**
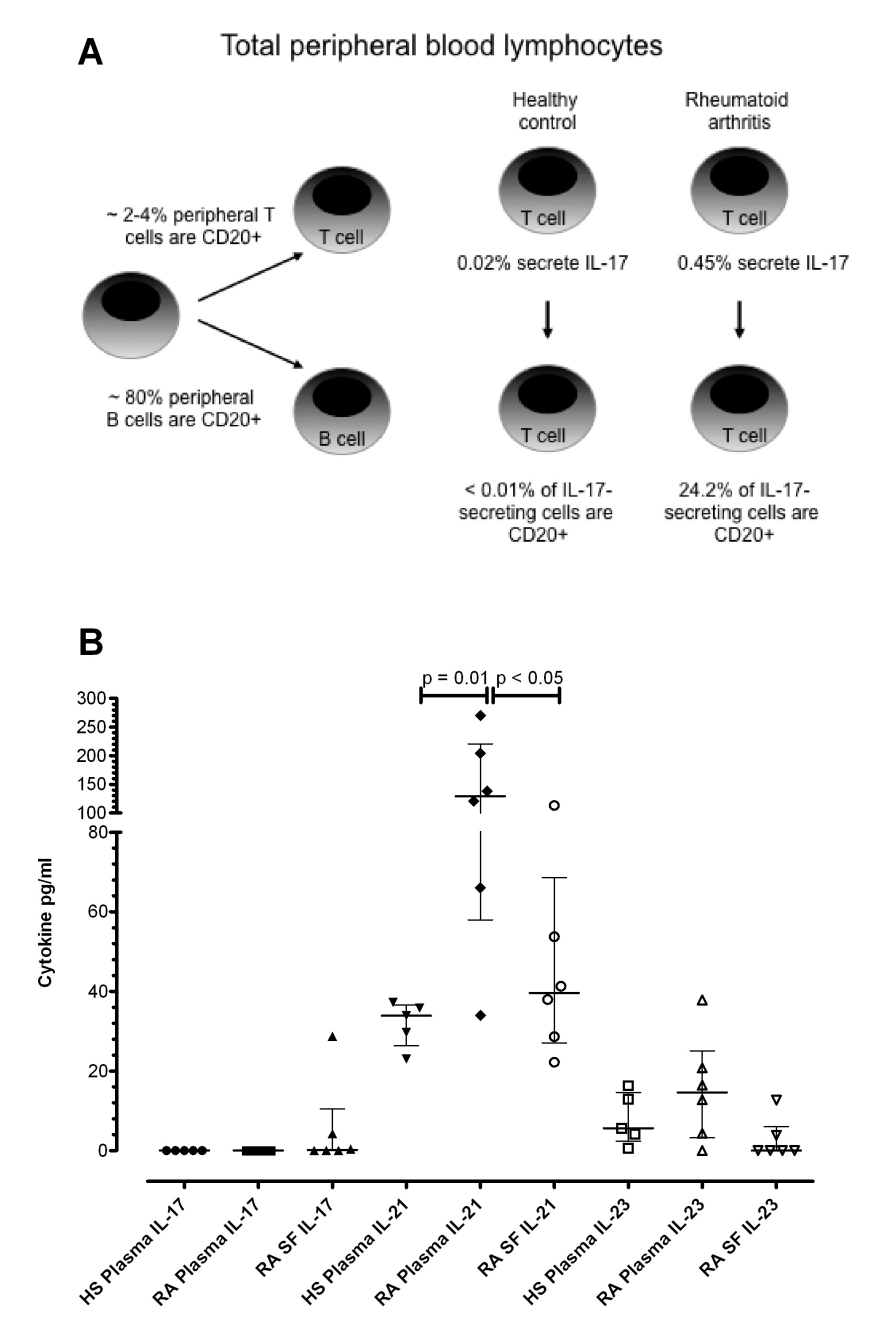
**Frequency of Th17 cells, and plasma IL-17, IL-21, and IL-23 concentrations**. **(A) **Schematic summarizing the proportions of CD20^+ ^and Th17/CD20^+ ^cell subsets in healthy subjects (HS) and rheumatoid arthritis (RA) patients. **(B) **Plasma and synovial fluid (SF) concentrations of IL-17, IL-21, and IL-23 as measured by enzyme-linked immunosorbent assay in RA subjects. The same cytokine concentrations were evaluated in the plasma of HS for comparison. IL, interleukin; Th, T helper cell.

As summarized in Figure 6A, approximately 2% to 4% of peripheral blood T cells were CD20^+ ^whereas 80% of B cells were CD20^+^. In healthy subjects, only 0.02% of their peripheral blood T cells secreted IL-17 and less than 0.01% of these IL-17-secreting cells were actually CD20^+^. In contrast, in the patients with RA, 0.45% of their total peripheral T cells secreted IL-17, of which 24.2% were CD20^+^. The detection of greater numbers of Th17 cells in RA patient blood upon stimulation with CytoStim suggests that CD20^+ ^and CD20^- ^Th17 cells are already developed and differentiated in higher numbers in RA patient blood. Thus, we measured the concentrations of IL-17 in the plasma (and SF where available). We also measured IL-21 and IL-23 concentrations, as both of these cytokines have been implicated in Th17 cell development [15,16]. We did not detect IL-17 in the plasma of our healthy subjects or in the methotrexate-treated RA subjects (Figure 6B) but did detect IL-17 in the SF of three out of six patients (4.40 ± 28.8 pg/mL, range of 0.41 to 28.7 pg/mL; *n *= 3). Since IL-21 and IL-23 may enhance the development of Th17 cells, these cytokines were measured in the plasma of healthy subjects and the plasma and SF of patients with RA. IL-21 levels were significantly higher in the plasma of RA patients compared with control plasma (129.3 ± 162.7 pg/mL and 33.9 ± 10.2 pg/mL, respectively; *P *= 0.01). IL-21 was also detectable in the SF of five of the RA patients screened above (39.6 ± 41.6 pg/mL), but this was significantly lower (*P *< 0.05) compared with the plasma IL-21 levels in the matched RA plasma samples. IL-23 was detectable in all plasma samples of healthy control and RA subjects (5.7 ± 12.3 pg/mL and 14.7 ± 21.8 pg/mL, respectively) and detected at low concentrations in only two out of six matched SF samples (Figure 6B).

## Discussion

Several anti-CD20 monoclonal antibody therapies are currently approved or in phase II/III trials to treat autoimmune disease [17]. These (RTX: Rituxan/MabThera, ocrelizumab: 2H7; and ofatumumab: Arzerra) are designed to bind to CD20 on the surface of B cells, resulting in their elimination. The exact cellular and molecular mechanism by which the binding of anti-CD20 monoclonal antibodies to B cells induces cell death is not fully understood [18]. This is reflected in the fact that a significant number of patients do not respond to B-cell depletion therapy [19-21]. The general assumption is that incomplete/non-responders are not effectively B cell-depleted, and a number of strategies to improve the elimination of CD20^+ ^B cells in resistant RA have been considered [22]. However, the observation that there are populations of CD20^+ ^T cells may lead to alternative explanations for the mechanism of action and incomplete response [9]. For example, it may explain, in part, why anti-CD20 biologics may provide beneficial effects in T cell-dominated autoimmune diseases such as multiple sclerosis [23]. Anti-CD20 therapy is designed to eliminate CD20^+ ^B cells from the peripheral blood circulation but has also been shown to eliminate CD20^+ ^T cells in patients with RA [9]. However, the clinical significance of this has not been studied in detail. An explanation for the small proportion of CD20^+ ^T cells in some patients with RA may be that exposure of CD20^+ ^T cells to RTX masks the CD20 surface antigen. Indeed, in Figure 1C, we demonstrated that pre-incubation of lymphocytes with RTX masks the CD20 antigen, which is no longer detectable by anti-CD20-FITC.

In our study, we confirmed the existence of CD20^+ ^T cells in the peripheral blood of healthy subjects and RA patients, an observation that was made in the early 1990s [24] and recently reported by Wilk and colleagues [9] in RA patients at the protein and mRNA levels. A novel finding in our study is that a significant proportion of CD20^+ ^T cells are also capable of secreting IL-17, particularly in the RA subjects studied. There is some variability in the reported values for the percentage of Th17 cells normally residing in human peripheral blood. Such variation may be a function of the assay method used and the IL-17 stimulant employed [14,25,26]. In our study, medians of 0.02% (range of 0.0% to 0.3%) and 0.45% (range of 0.0% to 1.2%) of PBLs isolated from healthy controls and RA subjects, respectively, secreted IL-17 after stimulation with CytoStim. In a previous study using the same assay and stimulant, IL-17 was produced by between 0.04% and 2% of T cells (*n *= 21) [14]. Other researchers have employed intracellular staining for IL-17 detection and observed different proportions of lymphocytes positive for IL-17 secretion. In a study by Shen and colleagues [26], Peripheral blood mononuclear cells from healthy control subjects and RA patients were stimulated with a combination of phorbol myristate acetate and ionomycin and this led to the detection of approximately 0.5% and approximately 1% IL-17^+ ^T cells, respectively, whereas another study demonstrated the presence of 1% of IL-17-secreting T cells in healthy blood [25]. Other studies have demonstrated 6% IL-17^+^/CD161^+ ^T cells [27]. These latter studies employed different stimuli and parametric analysis (mean ± standard deviation) and therefore are difficult to compare with our results, which were non-parametric in nature. The above variation indicates that, dependently of the assay used, there is a variation in the median or mean percentage of Th17 cells detectable in healthy control blood. Nevertheless, differences in the percentage of Th17 cells between healthy control blood and disease blood are a consistent finding independently of the assay used. In our study, we used a flow cytometric secretory assay to quantify actively secreting IL-17 subgroups of cells. To ensure that CD20^+ ^cells were not simply binding to IL-17 released extracellularly, we performed immunohistochemistry on permeabilized CD20^+ ^cells and confirmed that a proportion of these cells contained intracellular IL-17.

Since some autoimmune diseases targeted by T cells are predominantly CD4-mediated (for example, SLE [28]) or CD8-mediated (for example, multiple sclerosis [29]) or both CD4- and CD8-mediated (for example, RA [30]), we examined the ratio of CD20^+ ^CD4^+ ^and CD8^+ ^cells in a small sample of healthy control subjects (*n *= 4). Among the T-cell population, we found a greater number of CD8 cells expressing CD20. This observation contrasts with the study by Wilk and colleagues [9], who showed that similar numbers of CD4 and CD8 cells expressed CD20. This may be accounted for by donor variability. We further examined the CD4/CD8 phenotype of CytoStim-stimulated IL-17-secreting, CD20^+ ^T cells from a separate group of healthy subjects (*n *= 11). We found CD20^+^IL-17^+ ^T cells positive for CD8 or CD4 in equal numbers. However, given that there are approximately twice as many CD4 cells as CD8 cells in the peripheral blood T cell population, this would suggest that CD8 T cells do preferentially express IL-17.

A criticism of the previous study by Wilk and colleagues [9] was that CD3^+^CD20^+ ^cells may be an artefact of T-cell selection and may actually represent T-cell/B-cell doublets [10]. Wilk and colleagues [31] vigorously rejected this possibility. Another concern was the use of one type of flow cytometer to detect CD20^+ ^T cells [10]. To address this concern, we used two types of flow cytometers, as well as fluorescence activated cell sorting in combination with confocal microscopy. In our flow cytometry analyses, cells were gated either according to forward and side scatter (Accuri flow cytometer; BD Biosciences, San Jose, CA, USA) or according to side scatter and electronic cell volume/diameter, allowing one to distinguish cells as single cells on the basis of size (Quanta SC flow cytometer). Using these different instruments, we found, in agreement with Wilk and colleagues [9,31], that the CD20^+^/CD3^+ ^double-positive cells were singlets, which were dimly positive for CD20. In addition, single cells, sorted by using a high-speed cell sorter, were identified by confocal microscopy to be positive for the cell markers CD4 or CD8 and CD20. Thus, the CD20^+ ^cells analyzed here are, beyond a doubt, CD19^- ^T-cell singlets. In addition, Wilk and colleagues [9] demonstrated by real-time polymerase chain reaction that CD3^+ ^T cells were expressing low levels of CD20, but not CD19 at the mRNA level. In our study, the intensity of CD20 protein expression on the surface of CD3 cells was an order of magnitude lower compared with CD20 expression on B cells, and this would reflect the lower levels of the mRNA expression observed by Wilk and colleagues [9]. Moreover, the expression of CD20 on T cells appeared patchy and this has not been observed on this subset of lymphocytes. However, CD20 expression on B cells upon cross-linking with anti-CD20 is heterogeneous and is thought to represent a clustering of CD20 and other molecules such as Fas into patches [32]. It may be that cross-linking of anti-CD20 to CD20 on T cells leads to a similar heterogeneous localization of CD20 and other molecules into raft-like structures on the cell surface, which presents as a patchy expression of CD20.

Prior to the present study, it was unknown whether the CD20^+ ^T cells isolated from healthy subjects and RA peripheral blood or RA SF were able to secrete the pro-inflammatory cytokine IL-17, which is implicated in the pathology of RA [11]. Recently a greater frequency, and functionally active number, of Th17 cells have also been observed in SLE [28]. Th17 cells have been implicated in initiating inflammation in numerous autoimmune diseases. However, the mechanism of action of IL-17 and subsequent pathology will depend, in part, on the location of the Th17 cells. In the case of RA, Th17 cells have been reported in the peripheral blood but with less frequency in the joints [33], where Th1 cells appeared more abundant. In contrast, others have observed both Th17 and Th1 cells in the joints of RA patients in abundance [34]. This may be explained, in part, by the plasticity of Th17 cells, which are known to develop from Th1 cells in the presence of other pro-inflammatory cytokines [35].

Key findings of the current study are that Th17 cells are up to 45-fold more abundant in the peripheral blood of RA patients compared with healthy control subjects (Figure 4C, left panel) and that approximately 24% of these IL-17-secreting cells also have a CD20 phenotype (Figure 4C, right panel). A pool of potentially autoreactive T cells that is capable of secreting the pro-inflammatory cytokine IL-17 and that also expresses CD20 is found in the peripheral blood of patients with RA. Thus a relatively small but significantly increased number of Th17 cells is present in the peripheral blood and SF of RA patients [16] which can potentially drive both inflammatory and humoral responses in RA [36]. Furthermore, the development of Th17 cells is regulated by a number of cytokines [15] that are often abundant during active RA, providing further reasoning for monitoring the development and elimination of these pro-inflammatory cells. Elevated levels of IL-17 have been shown to correlate with joint damage [37] and may act as a biomarker of RA pathology [38]. IL-17, a signature pro-inflammatory cytokine released from Th17 cells and implicated in RA pathology [11], is also seen in a number of other conditions, including inflammatory arthritis [34] and ankylosing spondylitis [26]. The generation of Th17 cells and their distribution at sites of disease pathology are of concern, as IL-17 can promote expression of other pro-inflammatory cytokines and effectors (for example, IL-6, IL-32, and inducible nitric oxide synthase), enhance recruitment of neutrophils and monocytes to inflammatory sites, and promote joint degradation [11]. Despite observing a greater proportion of Th17 cells in RA peripheral blood compared with healthy subject blood, we were unable to detect endodgenous IL-17 in the plasma of healthy subjects or RA patients. This is not surprising as the blood samples used in this study were taken from methotrexate-treated patients. However, the CD20^+^/Th17 cells in the blood were capable of secreting IL-17 *in vitro*, and this is in agreement with others [26]. We were able to detect IL-17 in three of six RA SF samples examined from the same patients. This is consistent with the concept that, following migration to the synovial joint space, Th17 cells may actively secrete IL-17. In this regard, the finding of fourfold higher median concentration of IL-21 observed in the plasma of the RA patients compared with healthy subject plasma suggests that this cytokine may be important in promoting the proliferation of Th17 cells or may reflect the secretion of this autocrine cytokine by the greater number of Th17 cells that we observed in RA peripheral blood. A recent study has shown that IL-21 enhances Th17 cell proliferation [39] and our results are consistent with this possibility. The levels of IL-23 in the plasma of healthy subjects and RA patients were not significantly different, and IL-23 was dectable in only two of the six RA SF samples we evaluated. This is consistent with the recent studies of others who have observed low IL-23 levels in RA SF compared with plasma [40] or detected IL-23 in only about half of SF samples analyzed [41], making its role in Th17 development less clear [16].

'Blockade therapeutics' to inhibit IL-17 [42] may prove beneficial. However, anti-CD20 therapeutics already appears to relieve many of the symptoms provoked by IL-17 in T cell-mediated diseases such as RA. Our results would support the notion that anti-CD20 drugs such as RTX can also bind to CD20^+^/Th17 cells and possibly eliminate them before they release pro-inflammatory cytokines in the synovial joints. Recent evidence suggests that RTX treatment of patients with active RA leads to a reduction in IL-17 and IL-22 production *in vitro *[43]. This would support the possibility that anti-CD20 therapeutics have an unexpected role in influencing the development of Th17 responses.

## Conclusions

The identification of Th17^+^/CD20^+ ^cells in our study suggests that this population of cells is a possible target for anti-CD20 therapy. Our results suggest that the effect of anti-CD20 therapy on the depletion of CD20^+ ^Th17 cells should be monitored in clinical trials.

## Abbreviations

APC: allophycocyanin; CyQ: Cyquant; DAPI: 4',6-diamidino-2-phenylindole; FITC: fluorescein isothiocyanate; ICS: intracellular cytokine staining; IL: interleukin; IQR: interquartile range; PBMC: peripheral blood mononuclear cell; PE: phycoerythrin; RA: rheumatoid arthritis; RTX: rituximab; SF: synovial fluid; SLE: systemic lupus erythematosus; Th: T helper cell.

## Competing interests

The authors declare that they have no competing interests.

## Authors' contributions

PE, PGW and RCH designed the research, wrote the manuscript, and performed statistical analysis. EB and JMT and PE performed the, flow cytometry, and IL-17 secretion assays and revised the manuscript. MdB and WH performed confocal microscopy and cell sorting experiments. AK performed the cytokine assays and some IL-17 secretion assays. RCH and NJV selected patients according to American College of Rheumatology criteria and specific medication and organized the sample collection. All authors read and approved the final manuscript.
